# Estimating the number of HTLV-2 infected persons in the world

**DOI:** 10.1186/1742-4690-12-S1-O5

**Published:** 2015-08-28

**Authors:** Edward L Murphy, Olivier Cassar, Antoine Gessain

**Affiliations:** 1 Departments of Laboratory Medicine and Epidemiology/Biostatistics, University of California San Francisco, San Francisco, California, USA; 2 Blood Systems Research Institute, San Francisco, California, USA; 3 Institut Pasteur, Unité d'Epidémiologie et Physiopathologie des Virus Oncogènes, Département de Virologie, Paris, France; 4 UMR 3569, Centre National de la Recherche Scientifique, Paris, France

## 

The number of HTLV-1 infected persons in the world has recently been estimated to be at least at 5 to 10 million or perhaps higher due to unavailable data (Gessain and Cassar Front Microbiol 2012). We decided to undertake a similar estimation for HTLV-2 infection. A targeted review of the literature revealed HTLV-2 prevalence estimates in blood donors (as a surrogate for the general population) and specific recognized high prevalence subpopulations in different regions of the world. Because blood donors are selected for low risk of viral infection and good health, we multiplied their prevalence by a factor of five to estimate the population prevalence. Data from studies using sensitive and specific assays for HTLV-2 antibody were preferred. Population data were obtained from national censuses or, for certain high-risk groups, Google searches. For each subpopulation, prevalence was multiplied by the size of the population. We estimate that there are 800,000 (range 670,000 - 890,000) HTLV-2 infected persons globally. The largest number of such persons is in the United States (400,000-500,000) reflecting the confluence of endemic Amerindian, hyperendemic injection drug user (IDU) and secondary sexual spread to the general population. Brazil had the second-largest population of infected persons at 200,000-250,000 and a similar epidemiology. In Europe, where HTLV-2 infection is found almost exclusively among IDU, we estimate about 20,000-40,000 persons. Although they have the highest prevalence, other endemic Amerindian and Pygmy populations in Latin America and central Africa have very small populations and therefore contribute no more than 50,000-100,000 infected persons globally. In conclusion, the burden of HTLV-2 infection in the world is about 6 to 12 fold lower than that of HTLV-1. This represents a concentration of infection in specific high-risk groups and a lower degree of sexual and vertical transmission within the general population than for HTLV-1.

